# The influence of variations in background noise on Florida manatee (*Trichechus manatus latirostris*) detection of boat noise and vocalizations

**DOI:** 10.1371/journal.pone.0268513

**Published:** 2022-05-18

**Authors:** Athena M. Rycyk, Gordon B. Bauer, Randall S. Wells, Joseph C. Gaspard III, David A. Mann

**Affiliations:** 1 Division of Natural Sciences, New College of Florida, Sarasota, Florida, United States of America; 2 Division of Social Sciences, New College of Florida, Sarasota, Florida, United States of America; 3 Mote Marine Laboratory, Sarasota, Florida, United States of America; 4 Chicago Zoological Society’s Sarasota Dolphin Research Program, c/o Mote Marine Laboratory, Sarasota, Florida, United States of America; 5 College of Marine Science, University of South Florida, St. Petersburg, Florida, United States of America; Wildlife Conservation Society Canada, CANADA

## Abstract

A manatee’s primary modality to detect a vessel on a possible collision course is hearing as underwater visibility is limited in many manatee habitats and their visual acuity is poor. We estimate a Florida manatee’s ability to detect the sound of an approaching boat and vocalizations in four different soundscapes in Sarasota Bay, FL. Background noise samples were collected every 5 minutes for a two-week period during winter and summer at each location (2019 or 2020). Sound levels in third octave bands (0.5, 1, 2, 4, and 8 kHz) were measured and compared to manatee auditory hearing thresholds and to sound levels of an approaching boat traveling at a slow, medium, or fast speed. Background sound levels in a wider band (1–20 kHz) were calculated to model vocal communication space at each location. We found that a manatee’s estimated ability to detect an approaching boat differs greatly among locations, with time of day, and by season, and that fast boats are predicted to be detected later than slow boats. Latency of boat noise detection is estimated to sharply increase when considering unusually loud background noise levels. We suggest that such uncommonly loud conditions (e.g. 95^th^ percentile sound level), not just typical conditions (median sound level), are important to consider for understanding the problem of manatee-boat collisions. Additionally, background noise impacts estimated vocal communication space and may limit the ability of vocal-mediated mother-calf cohesion. Altogether, a manatee’s ability to detect acoustic signals of interest is expected to vary greatly spatially and temporally.

## Introduction

Marine soundscapes vary with biological (e.g., soniferous fish, invertebrates, and mammals), physical (e.g., tides, wave, and wind), and anthropogenic (e.g., boat noise and construction) activities over time and space [1–5]. Consequently, background sound level and frequency spectra are dynamic. Florida manatees (*Trichechus manatus latirostris*) inhabit shallow, coastal and inland waters that are shared with boat traffic and elevated ambient background noise can mask important signals such as the sound of an approaching boat and manatee vocalizations (“manatee” refers to Florida manatees unless otherwise stated) [6, 7].

Watercraft collisions account for a high number of manatee mortalities such that 564 manatees were killed by a watercraft collision between 2015 and 2019 [8]. As a threatened species under the U.S. Endangered Species Act, the high mortality rate from watercraft collisions is a major concern for the species [9]. Sublethal wounds from watercraft collisions are also common with an estimated 96% of adult manatees having scars from a collision, and a high percentage with scars from multiple collisions [10]. The sound of an approaching boat is a vital cue manatees use to avoid collisions [11]. In addition, boat noise may interfere with manatee vocal communication that is used to reflect motivational state, locate conspecifics, identify individuals, and facilitate mother-calf cohesion [12–15].

From controlled laboratory studies of hearing, we know manatees can hear from 0.25 to 76.1 kHz with best sensitivity from 6 to 32 kHz [16, 17]. Within this range of best sensitivity, critical ratios using continuous tones, which are a measure of susceptibility to masking, range from 18.3 to 46 [16, 18]. Additionally, their auditory temporal processing rate based on auditory evoked potentials is 600 Hz, which is midway between terrestrial mammals and dolphins [19]. Their relatively high auditory temporal processing likely contributes to their strong sound localization capabilities [20]. Altogether, their hearing capabilities, as measured under controlled laboratory conditions, are keen and suggest that they are able to detect the sound of an approaching boat and manatee vocalizations. What remains to be investigated is how their hearing capabilities fare in noisy soundscapes like those they encounter in the wild. Given the limited underwater visibility in many manatee habitats, hearing is likely the more important sense for manatees as sound travels effectively underwater and provides the earliest cue available that a boat is approaching.

Translating findings from controlled laboratory studies of hearing into real world situations manatees encounter in the wild requires considering background noise in the wild and propagation effects of sound transmission in shallow water habitats. We focus on predicting the impact of background noise on detectability of boat noise and manatee vocalizations as propagation effects in the study region, Sarasota Bay, FL, have been described elsewhere [21–23]. Evaluating real world scenarios also requires considering broadband sounds rather than the typical tone-based stimuli used in controlled laboratory studies [16]. Combining the tone-based hearing thresholds from controlled laboratory studies with recordings of boat noise, previously reported broadband source levels of manatee vocalizations, and real-world measurements of background noise allows us to estimate the impact of varying levels of background noise on a manatee’s ability to detect the sound of an approaching boat and manatee vocalizations.

Comparing tone-based hearing thresholds to broadband boat noise and background noise requires selecting appropriate bandwidths around tone frequencies to allow for comparisons. Critical ratios, which have been measured in manatees [16, 18], have been found to reasonably estimate critical bandwidth in some species [24], but not in others [25]. Third octave bands are commonly used to estimate auditory filter bandwidth [26–28] when a direct measurement is lacking. Comparing detection thresholds of narrowband signals (dB re 1 μPa) to third-octave bands (dB re 1 μPa rms), as is done in the current study, has been directly tested in harbor seals (*Phoca vitulina*) using the same animals in the same environment with the same equipment [29]. Above 200 Hz, the detection thresholds were similar. We use third-octave bands in the current study as they are comparable to other studies and one-third octave bands have similar scaling to mammalian critical bands.

We know that manatee hearing is sensitive enough to detect boat noise and vocalizations, but how detectability varies with background noise in their natural environment is not well understood. We characterize background noise at four locations in Sarasota Bay, FL, and compare it to 1) the sound of boats traveling at different speeds and estimate how much time a manatee has to react to an approaching boat traveling at different speeds and in different environments and 2) source level estimates of manatee vocalizations to estimate how background noise can impact manatee communication space.

## Materials and methods

### Sampling sites

**Fig 1:**
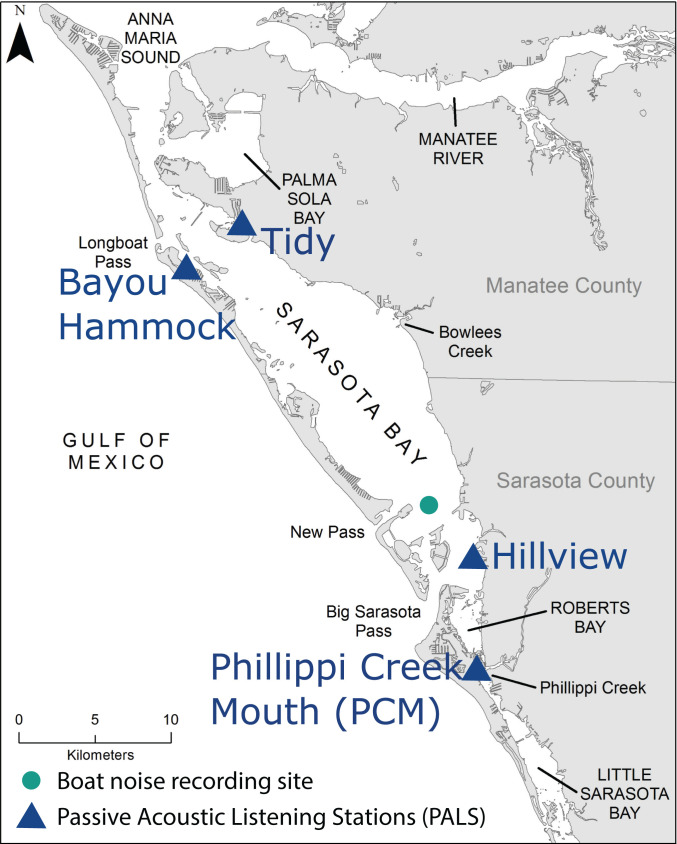
Map of Passive Acoustic Listening Stations (PALS) used in the current study (Sarasota Bay, FL). The four PALS stations used to record background noise are indicated by triangles. The boat noise recording site is indicated by a circle. The shapefile used for the map, Florida Shoreline (1 to 40,000 Scale), is available from the Florida Fish and Wildlife Conservation Commission GIS & Mapping Data Downloads (https://geodata.myfwc.com/) [33].

Four locations in the Sarasota Bay, FL, USA region were selected to represent areas manatees use and a variety of soundscapes manatees inhabit (Fig 1). The Tidy site (27.4509° N -82.6512° W) was located north of Tidy Island at the end of a dredged canal next to a series of boat slips. The water depth was 1.5 m and the bottom type was fine, silty mud. Boat traffic was minimal at this site with only local vessels occasionally entering the area at a slow speed. The Bayou Hammock site (27.4316° N -82.6764° W) was located east of Longboat Key in the entrance to Bishops Bayou. The hydrophone was in 1.5 m of water that sloped downward into an area dredged for local boat traffic with a wide (~250 m) seagrass meadow on the other side approximately 100 m from the hydrophone. Four hundred meters from the hydrophone on the other side of the seagrass meadow was a dredged channel, the Gulf Intracoastal Waterway, with heavy boat traffic. Boats traveling in the channel near the hydrophone generally travel slowly and boat traffic in the farther channel generally travels quickly. The Hillview site (27.3160° N -82.5447° W) was located on the east side of Sarasota Bay on the edge of a dredged boat channel. The water depth was 1.5 m with a sandy bottom extending to a seawall 9 m away. There was low boat traffic in the channel where the hydrophone was located; however, 220 m away the Gulf Intracoastal Waterway had much heavier traffic. A narrow (~80 m), shallow seagrass meadow separated the two channels. The Phillippi Creek Mouth (PCM) site (27.2712° N -82.5427° W) was located on Siesta Key at the intersection of a narrow constriction of the Gulf Intracoastal Waterway and Phillippi Creek. The water depth was 0.5 m on a slope to the Intracoastal Waterway with a sandy bottom. Boat traffic was heavy in this area, but vessel speed was restricted.

The soundscape at the Tidy site was mostly quiet with infrequent anthropogenic (boat) and biological noise (fish and snapping shrimp) relative to the other locations. The soundscape at the Bayou Hammock site included more boat noise than the Tidy site, but less than the Hillview and PCM sites. There was a richer biological contribution to the soundscape at this site with higher density of snapping shrimp snaps and fish-produced sounds. The Hillview soundscape also included biological noise but had a higher level of boat noise than the Bayou Hammock site. The highest occurrence of boat noise was at the PCM site and biological noise was less frequent compared to other sites. Manatees commonly use the Tidy, Bayou Hammock, and Hillview sites [31, 32]. The PCM site serves as a travel corridor for manatees passing between frequently used areas such as Robert’s Bay and an alcove off the Intracoastal Waterway approximately 700 m south of the PCM site [32].

### Background noise measurements

Land-based passive acoustic listening stations (PALS) were deployed at four locations in the Sarasota Bay, FL region to record underwater sound using HTI-96-MIN hydrophones (sensitivity -180 dBV/uPa, 44.1 kHz sampling rate; High-Tech Inc.) (Fig 1). The stations were deployed on private property (personal residences or a condominium) with permission of the owners. None of the deployment locations were in protected areas that would require a permit to deploy equipment. Each solar-powered station recorded continuously and stored a wav file with 16-bit resolution every 5 minutes. At the Bayou Hammock, Hillview, and PCM sites a 5-minute wav file was stored every 5 minutes. At the Tidy site a 1-minute wav file was stored every 5 minutes. On rare occasion, a sound clip was not successfully recorded and/or stored because of power limitations or technical problems. Sound clips were successfully recorded and stored 94% of the time. For more information about the acoustic recording setup, see Rycyk et al. (2020) [34]. Data were used from a contiguous two-week period in winter (January or February, 2019 or 2020) and a contiguous two-week period in summer (August, 2019 or 2020) from each location. A 1-s section from each wav file was used to calculate the background third-octave rms sound pressure level (TOL_BG_) in five third-octave bands in MATLAB (S1 Data) [30]. The five third-octave bands were selected because they had a center frequency, 0.5, 1, 2, 4, and 8 kHz, that corresponded to previously collected measurements of manatee hearing thresholds [16]. The 1-s sections were each at the halfway point of the wav file that was stored every 5 minutes. A short time window was selected as the sound level of boat noise, particularly from fast boats, changes quickly as a boat travels towards a receiver. Differences in TOL_BG_ for each third-octave band between locations, season (winter and summer), and time of day (07:00–18:59)/night (19:00–06:59) were evaluated by comparing median TOL_BG_ values and visually inspecting the TOL_BG_ distribution using violin plots [30, 35].

### Boat noise measurements

Boat noise recordings used the same boat (6.7 m length with a 225 HP Yamaha 4-stroke engine) traveling at three different speeds (7, 17.4, and 26.2 mph). The boat was driven at a constant speed by a researcher and passed the recording location within 1–2 m. The boat traveled in a 4 m deep channel outside a manatee idle speed zone (Fig 1). Recordings were collected during the day from an anchored boat in the same channel with all systems turned off. At the recording location, two hydrophones (Reson TC4013, sensitivity -212 dBV/uPa, 2 Hz-180 kHz response range with VP1000 amplifiers, 32 dB gain and a 100 Hz high pass filter) recorded the sound of the approaching boat. One hydrophone was 15 cm below the surface to approximate the depth of sound reception by a manatee breathing at the surface and the other hydrophone was 1 m below the surface to represent a typical manatee depth when not breathing at the surface. A manatee is at highest risk of a collision when at the surface but spends most of its time deeper in the water column therefore most of the time a manatee is exposed to boat noise it is deeper than 15 cm. Third-octave rms sound pressure level of the boat noise (TOL_BOAT_) was calculated every second in the same third-octave bands used for background noise measurements [30]. Analysis was restricted to 30 seconds before the boat’s closest point of approach (CPA) to the recording location until 3 seconds afterwards. Biological noise during the recording periods was very low compared to the four background noise sampling locations.

### Comparison of hearing, background noise, and boat noise

A manatee’s ability to detect the sound of an approaching boat was estimated by comparing their hearing detection thresholds under quiet controlled laboratory conditions, the sound level of an approaching boat traveling at different speeds, and background noise measured in the Sarasota Bay region. Specifically, tone-based audiogram thresholds (dB re 1μPa) in Florida manatees from Gaspard et al. (2012) were compared to background noise (TOL_BG_) and boat noise (TOL_BOAT_) in the corresponding third-octave band [16]. Tone-based thresholds can be compared to third-octave bands centered around the same frequency as the tone because third-octave bands were used to estimate auditory filter bandwidth [29]. Two levels of background noise were used for each location to evaluate both a typical (50^th^ percentile) and elevated (95^th^ percentile) level of background noise. The time at which TOL_BOAT_ exceeds both TOL_BG_ and the corresponding hearing threshold was the estimate of when the sound of the boat, within that third-octave band, becomes detectable to a manatee. The time from when a boat potentially becomes audible to when the boat reaches the manatee’s location was calculated for each boat speed (7, 17.4, and 26.2 mph) in each third-octave band within each location.

### Manatee communication space

A practical spreading loss model (15log_10_r, model choice explained below) was applied to select manatee vocalization source levels to estimate vocalization sound level as it travels from the source. Estimated sound level of a manatee vocalization as it traveled was compared to median levels of background noise at four locations to estimate propagation distance at each location (the distance at which estimated vocalization sound level and median background noise intersect).

Three vocalization source sound levels were used in modeling to represent a range of possible vocalization sound levels: mean-sd, mean, and mean+sd from Miksis-Olds and Tyack (2009) [36]. The source levels were from the most common type of vocalizations (chirps) reported in Miksis-Olds and Tyack (2009) [36]. Background noise (Sound Level_BG_ dB re 1 μPa rms in the 1–20 kHz band) was calculated for 1-s windows every 5 minutes at the locations and time periods described in the *Background Noise Measurements* section (S1 Data) [30]. A 1–20 kHz band was selected to approximate the hand-selected filtering (minimum-maximum vocalization frequency) used in Miksis-Olds and Tyack (2009) [36] to calculate manatee vocalization source level.

The current study uses a simple practical spreading loss model because measurements of transmission loss in similar environments have been found to generally be greater than would be predicted by a cylindrical spreading model, but less than a spherical spreading model [22, 23, 37]. Transmission loss measurements made in the same region as our study, Sarasota Bay, are approximated well by using a practical transmission loss model [22, 23]. Many factors that differ between locations affect transmission loss, such as sound speed, water depth, bottom type, and sediment attenuation [21, 38, 39]. However, even more sophisticated models of transmission loss, such as the Monterey-Miami Parabolic Equation model, that incorporate these factors can greatly over- or under-estimate transmission loss [21]. Using a model of practical spreading loss across our four locations agrees well with transmission loss experiments in the same region, provides consistency across locations to bolster generalizability of results, and allows for a straightforward estimate of detection distance. While estimated detection distance will vary between models of transmission loss and environments, the relationship between background noise and vocalization detection distance will remain the same.

## Results

### Background noise

**Fig 2:**
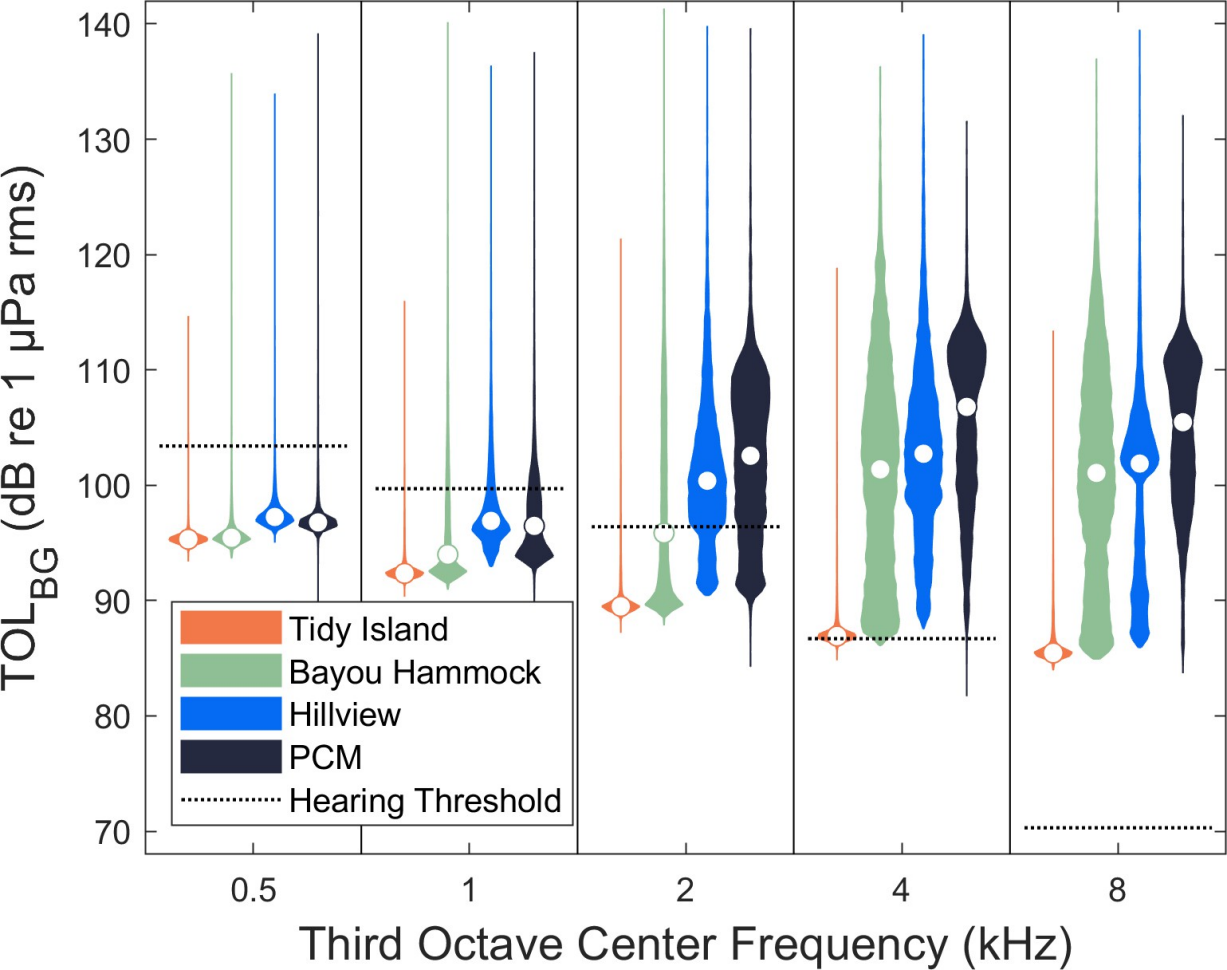
Background sound levels for 5 third octave bands at four locations. Third octave levels (TOL_BG_) in five bands for 1-s windows every 5 minutes (n = 30,226) at four locations in Sarasota Bay, FL. The white circle in each violin indicates the median value for that group. The dashed lines represent the tone-based manatee hearing threshold at each center frequency from Gaspard et al. (2012) [16].

**Fig 3:**
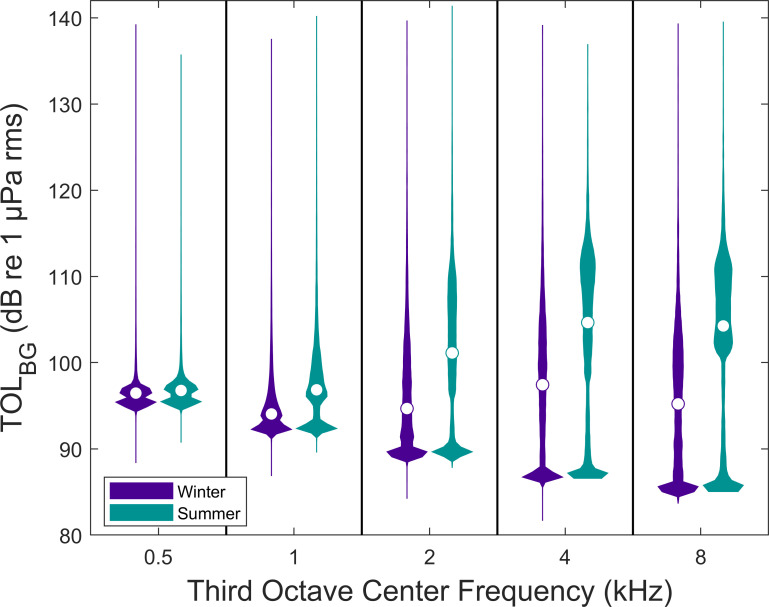
Background sound levels in winter and summer in third octave bands. Third octave levels (TOL_BG_) in five bands for 1-s windows every 5 minutes in winter (n = 15,208) and summer (n = 15,018). TOL_BG_ measurements include four locations in Sarasota Bay, FL. The white circle in each violin indicates the median value for that group.

**Fig 4:**
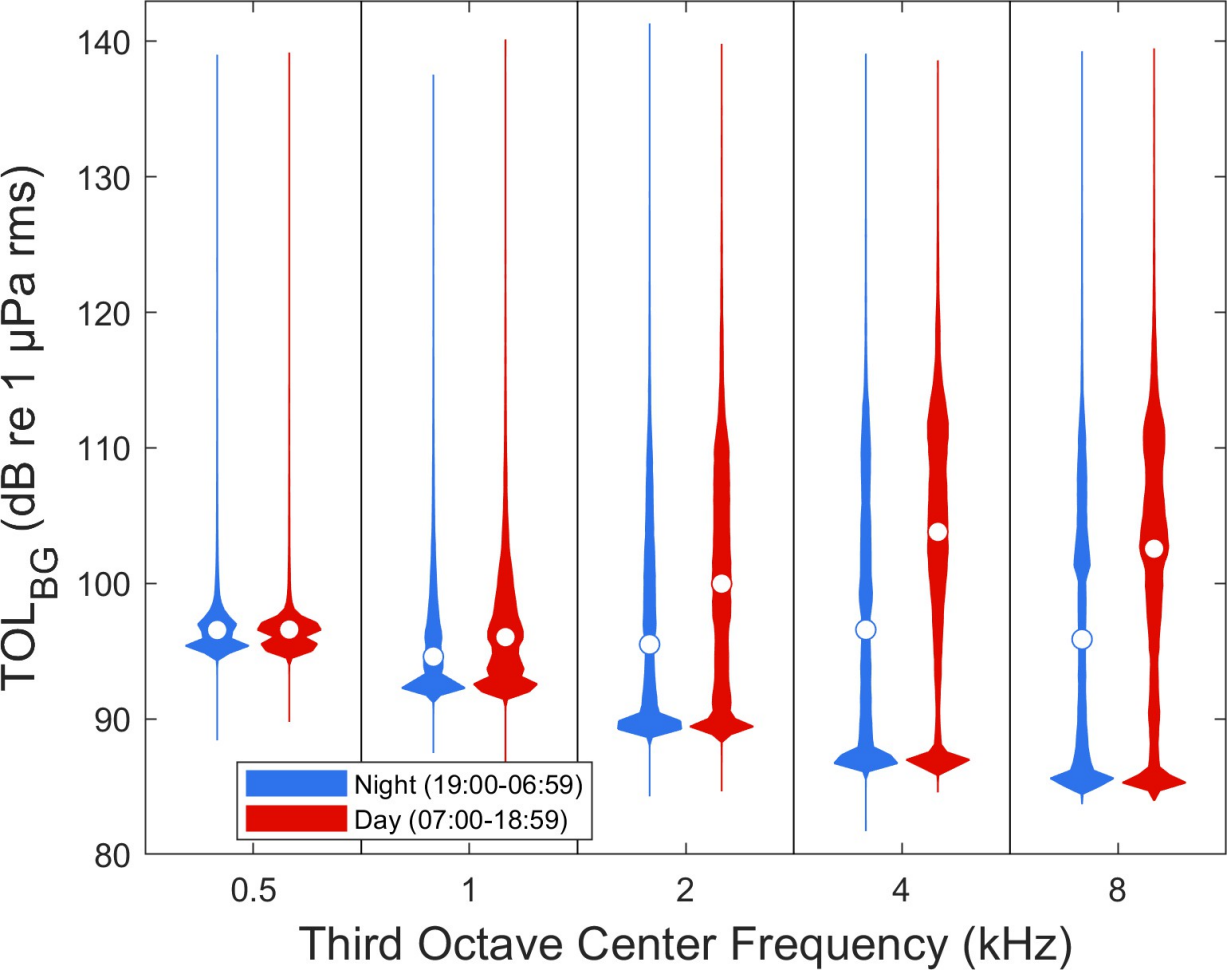
Background sound levels by time of day in third octave bands. Third octave levels (TOL_BG_) in five bands for 1-s windows every 5 minutes in different periods of the day. The time periods are day (07:00–18:59, n = 14,216) and night (19:00–06:59, n = 16,010). TOL_BG_ measurements include four locations in Sarasota Bay, FL. The white circle in each violin indicates the median value for that group.

TOL_BG_ differed among locations across the third octave bands calculated (0.5, 1, 2, 4, and 8 kHz) (Fig 2). The Tidy location (n = 6,386) was quietest followed by Bayou Hammock (n = 8,062). Hillview (n = 7,994) and PCM (n = 7,784) were louder than both Tidy and Bayou Hammock. Hillview was louder than PCM in the 0.5 and 1 kHz bands, but quieter in the 2, 4, and 8 kHz bands. TOL_BG_ in each third octave band was higher during the summer than during winter (Fig 3). TOL_BG_ in each third octave band was higher in the day than at night (Fig 4).

### Comparison of hearing, background noise, and boat noise

**Fig 5:**
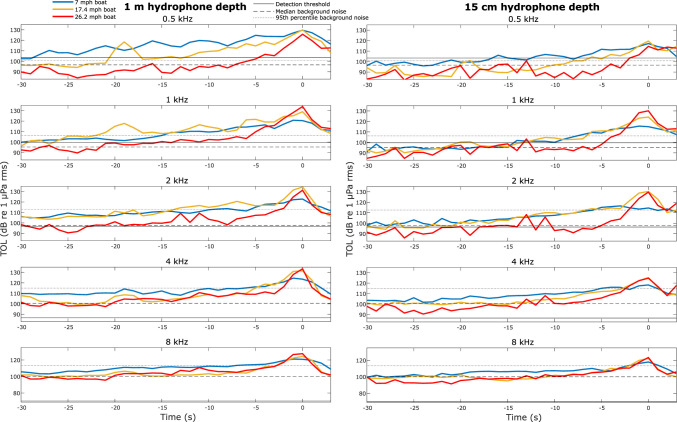
Detectability of boat noise at different depths. A comparison of boat noise recorded at two depths (left column boat recordings at 1 m depth and right column boat recordings at 15 cm depth), and background noise in five third octave frequency bands (top to bottom: 500 Hz, 1 kHz, 2 kHz, 4 kHz, 8 kHz). The sound level (TOL_BOAT_) of three boat passes (blue line at 7 mph, orange line at 17.4 mph, and red line at 26.2 mph) using the same boat are shown in each plot. The boat’s closest point of approach (within 2 m of the hydrophone) occurred at 0 seconds in all plots and negative time indicates the number of seconds before the boat’s closest point of approach. Background noise levels (TOL_BG_) measured across all four stations are represented by a dashed line for the 50^th^ percentile value and a dotted line for the 95^th^ percentile value. The detection threshold for each third octave center frequency from Gaspard et al. (2012) [16] is represented by a solid line.

Manatee detection thresholds in quiet controlled laboratory conditions were below median background noise levels across all four stations (pooled) for the 2, 4, and 8 kHz third octave bands (Fig 5). The detection threshold was above the median background noise level across all four stations (pooled) for the 1 kHz third octave, but below the 95^th^ percentile background noise level. The 500 Hz detection threshold was higher than the 95^th^ percentile background noise level across all four stations (pooled). TOL_BOAT_ was lower across third octave bands when recorded at a depth of 15 cm versus 1 m depth (Fig 5). The lower TOL_BOAT_ is expected to correspond to a delay in a manatee’s ability to detect an approaching boat (TOL_BOAT_ rises above TOL_BG_ and the detection threshold later relative to the boat’s closest point of approach).

**Fig 6:**
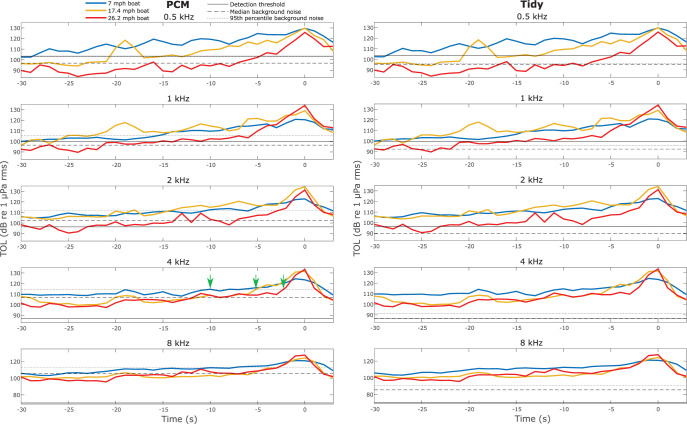
Detectability of boat noise at a noisy and quiet location. A comparison of boat noise recorded at two locations (left column PCM and right column Tidy), and background noise in five third octave frequency bands (top to bottom: 500 Hz, 1 kHz, 2 kHz, 4 kHz, 8 kHz). The sound level (TOL_BOAT_) of three boat passes (blue line at 7 mph, orange line at 17.4 mph, and red line at 26.2 mph) using the same boat are shown in each plot. The boat’s closest point of approach (within 2 m of the hydrophone) occurred at 0 seconds in all plots and negative time indicates the number of seconds before the boat’s closest point of approach. Background noise levels (TOL_BG_) measured are represented by a dashed line for the 50^th^ percentile value and a dotted line for the 95^th^ percentile value. The detection threshold for each third octave center frequency from Gaspard et al., 2012 [16] is represented by a solid line. Green arrows on the PCM 4 kHz plot indicate the earliest time the sound level of each boat crosses the 95^th^ percentile background noise level in that band.

**Fig 7:**
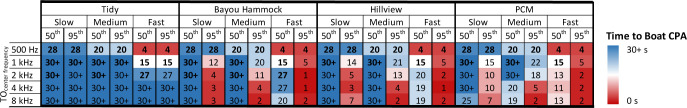
Estimated time a boat is detectable at four locations using three boat speeds. The earliest time (s) to a boat’s closest point of approach (CPA) in which TOL_BOAT_ is greater than TOL_BG_ and the detection threshold (from Gaspard et al., 2012 [16]) in the respective third octave band (TO_center frequency_). This calculation is displayed for four locations in Sarasota Bay, FL (Tidy, Bayou Hammock, Hillview, and PCM) using three boat speeds (slow = 7 mph, medium = 17.4 mph, and fast = 26.2 mph), and two values of TOL_BG_ (50^th^ and 95^th^ percentile for the respective location). Examples of the earliest time the sound level of each boat crosses the 95^th^ percentile background noise level in the 4 kHz band are represented by green arrows in Fig 6 for the PCM location. Bolded values indicate that detection is limited by the detection threshold (i.e. the detection threshold is higher than the TOL_BG_ being considered) and unbolded values are limited by background noise (i.e. TOL_BG_ is higher than the detection threshold). The time to a boat’s CPA may serve as a proxy for how long a manatee has to respond to an approaching boat before it reaches the manatee and is color-coded such that red values indicate very little time for a manatee to respond.

An approaching boat is expected to not be detectable (TOL_BOAT_ rises above TOL_BG_ and the detection threshold) until much later at locations with higher background noise (PCM) than quieter locations (Tidy) (Figs 6 and 7). A fast boat would not be detectable until much later than a boat traveling at a medium or slow speed (Fig 7).

### Communication space

**Fig 8:**
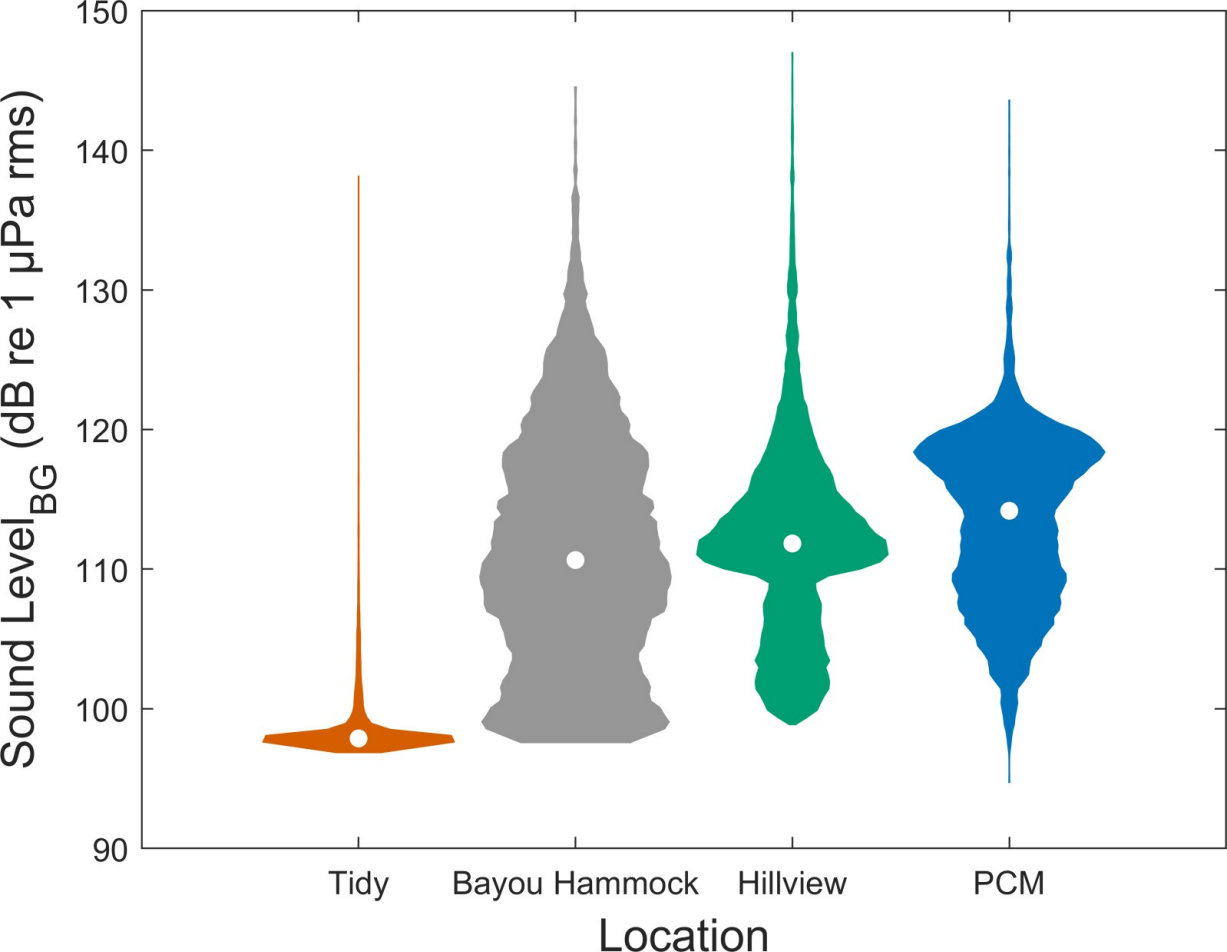
Background sound levels at four locations for a frequency band used in manatee communication. Sound levels_BG_ (dB re 1 μPa rms) in the 1–20 kHz frequency band by location. Sound level samples were collected every 5-min and calculated for a 1-s time window (Tidy n = 6,386, Bayou Hammock n = 8,062, Hillview n = 7,994, and PCM n = 7,784). The white circle in each violin indicates the median value for that location.

**Fig 9:**
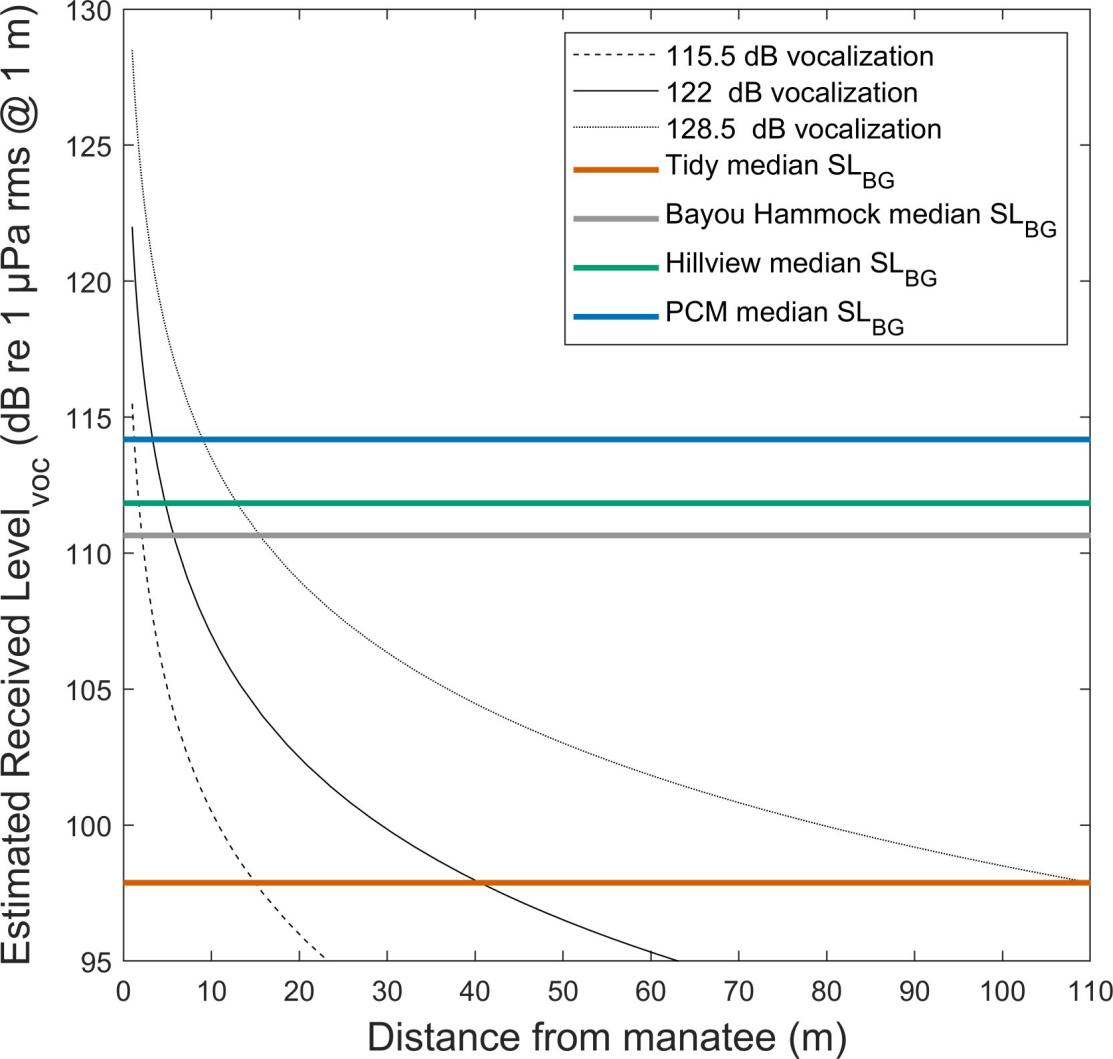
Estimated detection distance of a manatee vocalization at four locations. Estimated received sound level of a manatee vocalization by distance. Transmission loss is estimated using a practical spreading loss model (15log_10_r). Three source levels of manatee vocalizations are included (115.5 dB is the dashed line, 122 dB is the solid line, and 128.5 dB is the dotted line). The median SL_BG_ (Sound Level_BG_) at four locations (1–20 kHz band) are represented by color-coded horizontal lines.

Background noise in the 1–20 kHz band (Sound Level_BG_) varied among the four locations with PCM being the loudest location followed by Hillview, Bayou Hammock, and then Tidy (Fig 8). Accordingly, estimated manatee vocalization propagation distance varied across locations (Fig 9 and Table 1). Vocalizations would propagate farthest at the Tidy location. Estimated propagation distance varies greatly with background noise (Sound Level_BG_), environmental features, and vocalization source level.

**Table 1:** Estimated manatee vocalization detection distance at four locations using three source levels.

	Tidy	Bayou Hammock	Hillview	PCM
**Distance (m) [SL**_**VOC**_ **115.5 dB]**	15.0	2.1	1.8	1.2
**Distance (m) [SL**_**VOC**_ **122 dB]**	40.6	5.7	4.8	3.3
**Distance (m) [SL**_**VOC**_ **128.5 dB]**	110.0	15.5	12.9	9.0

Estimated detection distance (m) for a manatee vocalization using a practical spreading loss model (15log_10_r). Three vocalization source levels (SL_VOC_) are used and the values of the intersection of the model with the median SL_BG_ at each location are used as an estimation of propagation distance.

## Discussion

### Background noise and boat speed

We found large variations in background noise among locations, time of day, and season that were estimated to influence a manatee’s ability to detect the sound of an approaching boat. This suggests that the time available to a manatee to respond to an approaching boat varies with location, time of day, and season. More so, background noise may vary greatly within a location (Bayou Hammock, Hillview, and PCM) and over time (season and time of day). The most striking difference is between a consistently quiet location away from boat traffic (Tidy) and the comparably louder locations near boat traffic. The higher background noise during the day and summer when more boats are on the water also suggests that boat noise is an important contributing factor to the increased background noise at Hillview and PCM, and to a lesser extent Bayou Hammock.

From a manatee’s perspective, consider that a boat traveling at a fast speed may be detectable 27 seconds in advance at Bayou Hammock under typical (median) conditions, but would be detectable only 5 seconds in advance with elevated (95^th^ percentile) background noise (Fig 7). In both scenarios, the manatee is at the same location but is provided with a shorter warning of an approaching boat when background noise is elevated. Boats traveling at a slow or medium speed with a typical (median) background noise level, across locations, are estimated to provide ample time to respond (minimum of 30 seconds). However, elevated background noise is expected to sharply curtail this time window. This suggests that considering high percentile background noise levels can identify scenarios that are more dangerous for manatees.

In addition to background noise level, boat speed greatly impacts the estimated time window a manatee has to respond to an approaching boat. For example, at the PCM location, a slow boat may be detectable more than 30 seconds in advance with a typical (median) background noise level (28 seconds with a 95^th^ percentile background noise level) while a fast boat may be detected only 15 seconds in advance with a typical (median) background noise level (5 seconds with a 95^th^ percentile background noise level). The sharp difference in estimated detectability of a slow versus a fast boat relates to its distance to the manatee. A slow-traveling boat would be detectable earlier because it is closer to the manatee. For example, 15 seconds before a boat traveling 7 mph reaches a manatee it is 47 m away, whereas a boat traveling 26.2 mph is 176 m away. Therefore, the slow boat that is producing lower amplitude sound is estimated to be louder at the manatee’s location because of its proximity. Field observations confirm that manatees change their behavior earlier when a boat is traveling slowly [40].

We focused on the impact of background noise on the detectability of boat noise. However, variation in detectability of the sound of an approaching boat is further complicated by the Lloyd Mirror effect creating interference with sound propagation when the manatee is near the water’s surface [41, 42]. The sound of an approaching boat is predicted to not be detectable until much later when the sound receiver (hydrophone or manatee) is close to the surface (15 cm compared to 1 m). Unfortunately, a manatee is at a greater risk of a boat collision when it is close to the surface and within the depth of a boat’s hull and/or propeller.

The greater attenuation of a boat’s sound at the surface may not translate into a delayed response by the manatee. During a study of wild manatee behavioral response to approaching boats, a manatee’s probability of responding and the timing of such a response was not influenced by the manatee’s depth in the water column [40]. Perhaps a manatee can adjust for delayed detection at the surface by being more responsive once a boat is detected or by cueing into the different frequency spectra of a sound impacted by the Lloyd Mirror effect. Another possibility is that the Rycyk et al. (2018) study did not detect a difference in a manatee’s behavior based on its depth due to their sample only including a small number (n = 29) of close boat approaches (within 10 m) or not measuring an important covariate outside of the 13 factors included [40].

In addition to interactions with the water’s surface, bathymetry contour and bottom type influence sound propagation [41, 43]. The bathymetry contour of shallow coastal waters is complex with shallow seagrass meadows, deep dredged channels for boat traffic, seawall-lined canals, and passes that open into larger bodies of water. The variations in depth and angle of slopes between areas of different depth can drastically change the rate of transmission loss as sound travels. For example, sound traveling upslope from a deeper channel onto a shallow area can become concentrated in the smaller volume of water but is also subjected to more boundary interactions because of the shallower depth [43]. Another example are narrow channels, such as canals and the intracoastal waterway, that may act like a duct that reduces loss from interactions with the bottom, especially if sound propagation is in the direction of a downslope [43]. The type of sediment and presence of vegetation on the bottom further complicates sound transmission. The effect of bottom type on sound propagation depends largely on the ratio of sound speed in the water relative to the sediment. Sound speed in sediment is influenced by a variety of factors such as sediment type, size, density, air content, porosity, and bioturbation [44–46]. Seagrass on top of the bottom can affect sound propagation depending on seagrass species, density, and bubble production [47–49]. Regardless of bathymetry contour and bottom type, elevated levels of background noise can mask boat noise thereby limiting a manatee’s ability to detect the sound of an approaching boat.

Water depth is another important factor when considering shallow water acoustics as shallow water can inhibit propagation of low frequency sound because it cannot accommodate the wavelength [41]. The boat noise recordings were collected in a 4 m deep channel to minimize the impact of water depth limiting sound propagation of low frequencies. A water depth of 4 m can accommodate frequencies above approximately 375 Hz. The acoustic impedance of the bottom type is also an important consideration as a bottom type with similar acoustic impedance to water can expand the depth available for propagation of low frequencies [41]. If a manatee and an approaching boat are both in the same deep channel, such as at the boat noise recording site, then low frequency sound cues of the approaching boat would be available to the manatee. If the manatee, boat, or the path between them is shallow, then this information is removed. For example, if a manatee is in an area with a water depth of 1.5 m with a bottom type dissimilar in acoustic impedance to water, then the 1 kHz and lower components of boat noise would not be available for a manatee to detect. However, manatee hearing thresholds decrease for frequencies above 1 kHz [16]. This means manatees are better able to detect sound in the 2, 4, and 8 kHz range. Even though there is less energy in boat noise at these frequencies, manatees are better equipped to detect it. Even if depth limitations on the propagation of low frequency components of boat noise prohibit a manatee from detecting lower frequencies, we still predict a sharp distinction in the amount of time a manatee has to respond to an approaching boat based on background noise and boat speed based on higher frequency information (e.g. 2, 4, and 8 kHz in Fig 7). Manatees are commonly found in both deeper, dredged channels and shallow seagrass meadows and both habitats present challenges [31, 32, 50]. Deeper, dredged boat channels that allow for low frequency sound cues of an approaching boat are also where boat density is highest, and boats generally travel fast therefore manatees are at a higher risk of a collision and sustaining a more serious injury if they are hit. Shallow seagrass meadows tend to host less boat traffic and the boats are more commonly traveling slowly but low frequency sound cues can be stripped by the shallow depth and manatees may have more limited options to avoid an approaching boat because of depth limitations. Depth-limited propagation of low frequency sound adds another layer of complexity when predicting a manatee’s ability to detect the sound of an approaching boat and assessing risk of a manatee-boat collision, however the overall trends in variation of detectability because of boat speed and temporal and spatial variations in background noise would still be supported.

An important consideration when applying these results is that the detection thresholds used in the current study were determined from a behavioral audiogram of one manatee under human care (Buffett) [16]. The second manatee in the same study (Hugh) had a higher detection threshold for 0.5, 1, 2, and 8 kHz and a lower threshold for 4 kHz [16]. In Gerstein et al. (1999), behavioral tests of tone-based hearing thresholds of two other manatees used slightly different frequencies (0.5, 1.6, 3, and 6 kHz) than in the current study and therefore cannot be directly compared [17]. However, thresholds for similar frequencies (e.g., 3 and 4 kHz) were generally lower in the Gerstein et al. study [17].

The detection of sounds received against background noise, such as broadband sounds produced by boat engines, is complex. Physical factors (e.g., power of boat noise relative to background sounds, the relative directionality of sound sources, and distribution of frequencies), sensory factors (e.g., critical bandwidths and auditory frequency sensitivity), and other cognitive factors (attention and learning history) affect manatee perception and response to boat noise (physical and sensory factors are reviewed in [6, 7, 51]). Unfortunately, there is a paucity of research on these factors necessary to understand the effect of noise on manatee behavior. For example, peer-reviewed published studies of masking in manatees critical for understanding perception of broadband boat noise have typically tested tonal sounds [16]. Although we were able to estimate broadband hearing by using third octave bands, direct tests of masking of broadband sounds would be prudent. We estimated the time of a manatee detecting the sound of an approaching boat based on when the sound of the boat is greater than the background noise, but the sound of the approaching boat may need to be significantly louder than the background noise to be detected [18]. However, the broadband nature of boat noise means it spans multiple bands and this may facilitate a manatee’s ability to detect it. Other cognitive factors important to detection of boat noise such as attention, learning history, and decision making under the stress of multiple boats have not been investigated to our knowledge.

Our measurements of background noise include sound produced by boats in the area to capture realistic conditions. High TOL_BG_, such as the 95^th^ percentile examples used, likely represent periods with boat noise. Therefore, predicted detection of our boat noise measurements with high background noise represents the ability to detect the sound of an approaching boat when other boats are in the area. In psychophysical studies this would typically be done by measuring a just-noticeable difference in sound levels. This scenario is important to consider as manatees are commonly exposed to the sound of overlapping boat noise [40]. A manatee’s ability to detect the sound of an approaching boat can be reduced by the noise produced by other boats. Even if the sound of the approaching boat is louder than background boats it may be difficult to distinguish among boats and identify if one of them poses a threat. Additionally, overlapping boat noise may interfere with a manatee’s ability to localize the sound of a specific boat that poses a threat. Psychophysical tests of a manatee’s ability to localize a sound source demonstrated the ability to localize tonal and broadband sounds using an eight-choice discrimination setup [20]. However, only one sound source was presented at a time, and they did not assess a manatee’s ability to localize a particular sound source when there are multiple sources.

In the wild, reports of manatee responsiveness to the sound of an approaching boat varies greatly. How often manatees change their behavior when there is the sound of an approaching boat includes reports of 6%, 49%, 64%, and 89% of the time [11, 40, 52, 53]. Differences in reported responsiveness may be attributable to many factors including different definitions of a behavioral response, methods of observing and/or measuring responses, manatee populations, individual manatee history of exposure to boat noise, experimental design, and boat type, engine, and speed. We can now add to the list that differences in temporal and spatial variation in soundscapes may also contribute to the wide range in responsiveness reported. Additionally, manatees in different areas and time periods can have strikingly different time windows to detect and respond to an approaching boat. Further, multiple boats in an area, a common occurrence in Florida, can mask the sound of one another. A study of Antillean manatees (*Trichechus manatus manatus*) in a comparatively quiet location with less boat traffic (Belize) found manatees responded earlier and in a more pronounced manner to approaching boats compared to subsequent studies with Florida manatees where boats are more prevalent [40, 54].

### Communication space

Manatee vocalization detection distance estimates were found to be limited by background noise based on a practical spreading loss model. Background noise in a frequency range (1–20 kHz) that overlaps with manatee vocalizations, which includes the range of greatest hearing sensitivity [16, 17], varied between and within locations. In particular, the Tidy location was quietest and exhibited the least variation in background noise (Fig 8). At this location, vocalizations can potentially be detected from farther away, allowing manatees to communicate over a greater distance. The other locations (Bayou Hammock, Hillview, and PCM) are estimated to have very limited communication space under typical (median) background noise conditions. The source level of manatee vocalizations greatly influences the estimated detection distance such that a 13 dB increase at Tidy results in a 7.3 times greater detection distance. Given the sensitivity of detection distance to source level, it is worth closely considering the methods used to estimate source level.

Previously reported sound levels of Florida manatee vocalizations may be underestimates of source levels. First, manatees may naturally produce quiet vocalizations in quiet locations. Indeed, the quietest source level estimates of vocalizations were obtained in freshwater areas that likely had low ambient noise compared to marine environments [55, 56]. Miksis-Olds and Tyack (2009) found evidence that manatees can adjust their vocalizations, including source level, as a function of ambient noise levels [36]. Second, some source level estimates were obtained from large groups of manatees. Manatees in close proximity may not need to vocalize loudly to communicate with conspecifics, and flexibility of vocalization source level based on behavioral context was found in manatees by Miksis-Olds and Tyack (2009) [36]. In another sirenian species, dugongs (*Dugong dugon*) increase the source level of their vocalizations when a speaker playing dugong vocalizations is farther away [57]. Another reason group size is important to consider is that a large group size when measuring source level may attenuate the vocalizations before reaching the hydrophone. The sound path between a vocalizing manatee and the recording hydrophone may be occupied by many manatee bodies. Such interference can cause a decrease in sound level as was observed in a sound localization study with captive manatees in which body shadowing attenuated signals [20]. Attenuated vocalizations would translate into an underestimate of source level. Third, directivity of manatee vocalizations is unknown. The path of vocalization propagation from the source of sound production, the larynx, to the external environment has not been confirmed but is suspected to travel through the nasal cavity, floor of the mouth, or throat [58, 59]. The directionality of production and transmission of manatee vocalizations is unknown, but if it is directional, then the intensity of a vocalization would vary depending on the orientation of the sound receiver (e.g., a hydrophone) relative to the manatee, and vocalizations recorded off-axis would be quieter. Under this scenario, mean source level estimates obtained irrespective of manatee orientation would be an underestimation. Such directionality of vocalization production has been found in other marine mammals to varying degrees [60, 61]. Source levels of dugong vocalizations estimated from recordings obtained directly in front of temporarily restrained wild dugongs were on average much higher (139 dB re 1 μPa at 1m) than those reported for Florida manatees [62]. The large range in source level estimates between and within studies suggests manatees can alter the source level of their vocalizations and/or have directivity in vocalization production [56]. A greater understanding of manatee vocalization source level flexibility and directionality would be beneficial to improving our understanding of manatee communication.

Controlled laboratory recordings of manatee vocalizations from differing axes and in varying levels of background noise would complement the previously collected field measurements. Another consideration is the type of measurement used to characterize the sound level of manatee vocalizations. Typically, it is reported as rms sound level for a wide bandwidth. However, manatee vocalizations are commonly organized as a series of harmonic narrow bands. There could be significant energy in each narrowband for a manatee to detect but this would be diluted by calculating a rms sound level for a wide range of frequencies. Regardless of the source level estimates, communication space is expected to vary greatly with background noise level, and therefore, vary temporally and spatially.

The ability of background noise to mask manatee vocalizations may be partially mitigated by compensation mechanisms. Other marine mammal species have been found to compensate for elevated background noise by increasing the amplitude of their vocalizations (Lombard effect), shifting the frequency of their vocalization, changing vocalization structure, increasing duration of vocalizations, and vocalizing more often [6, 63–69]. Manatees can also vary the structure, duration, sound level, and rate of vocalizations in relation to behavioral context and ambient noise levels [36]. The extent to which these mechanisms can compensate for elevated background noise is unclear. Another consideration is that manatee habitat choice influences how background noise and their vocalizations propagate in their vicinity. Among environments manatees utilize, transmission loss varies and manatees prefer seagrass meadows with higher transmission loss in the dominant frequency range of their vocalizations [21]. Higher transmission loss creates a quieter environment that manatees may prefer when selecting a seagrass meadow.

Vocal communication is important for manatees to convey motivational state and maintain contact, especially for mothers and calves [12, 13]. Manatees can likely recognize individuals based on individually-distinct properties of their vocalizations [12–15, 70]. The impact of background noise masking manatee vocal communication is unknown but could include limiting mother-calf cohesion. As calves venture away from their mother to explore their environment, vocalization duets between the mother and calf facilitate their reunion. If background noise limits the range of vocal communication, then mothers and calves may need to spend more time and energy to find one another or may be unable to reunite at all. Calves are dependent on their mothers for 1–2 years and an average of 15 orphaned calves are rescued yearly in Florida (2016–2020, includes preliminary data) [71, 72]. Manatee calves can be orphaned when the mother dies; however, in some cases the cause of the mother’s absence is unknown and may occur because of separation. Louder background noise is predicted to reduce communication space and therefore limit vocally-mediated cohesion between mother’s and calves. The predicted temporal and spatial variations in communication space caused by differing background noise could impact habitat selection for mothers and is an important consideration for future studies.

## Conclusions

In conclusion, temporal and spatial variations in background noise impacts a manatee’s estimated ability to detect boat noise and vocalizations. In particular, high levels of background noise (95^th^ percentile) strongly decrease a manatee’s estimated ability to detect boat noise, and we suggest considering not just typical conditions (median) but also high levels of background noise when evaluating a manatee’s ability to respond to an approaching boat. Boat speed is estimated to strongly influence the time a manatee has to respond to an approaching boat such that manatees are expected to detect the sound of an approaching boat earlier if the boat is traveling slowly. High background noise levels, created in part by boats, are predicted to limit a manatee’s ability to communicate vocally by decreasing the distance vocalizations can travel.

## Supporting information

S1 Data Sound level measurements used in analyses. (CSV)
